# Role of protein Post-translational modifications in enterovirus infection

**DOI:** 10.3389/fmicb.2024.1341599

**Published:** 2024-02-26

**Authors:** Xiaohui Zhao, Yibo Hu, Jun Zhao, Yan Liu, Xueman Ma, Hongru Chen, Yonghua Xing

**Affiliations:** ^1^Department of Pathogen Biology, School of Medicine, Qinghai University, Qinghai, China; ^2^Department of Orthopaedic Trauma, The Affiliated Hospital of Qinghai University, Qinghai, China; ^3^Department of Immunology, School of Medicine, Qinghai, China; ^4^Department of Traditional Chinese Medicine, School of Medicine, Qinghai University, Qinghai, China; ^5^Department of Public Health, School of Medicine, Qinghai University, Qinghai, China; ^6^Department of Genetics, School of Medicine, Qinghai University, Qinghai, China

**Keywords:** enterovirus infection, post-translation modification, host factors, pathogenesis, enterovirus life cycle

## Abstract

Enteroviruses (EVs) are the main cause of a number of neurological diseases. Growing evidence has revealed that successful infection with enteroviruses is highly dependent on the host machinery, therefore, host proteins play a pivotal role in viral infections. Both host and viral proteins can undergo post-translational modification (PTM) which can regulate protein activity, stability, solubility and interactions with other proteins; thereby influencing various biological processes, including cell metabolism, metabolic, signaling pathways, cell death, and cancer development. During viral infection, both host and viral proteins regulate the viral life cycle through various PTMs and different mechanisms, including the regulation of host cell entry, viral protein synthesis, genome replication, and the antiviral immune response. Therefore, protein PTMs play important roles in EV infections. Here, we review the role of various host- and virus-associated PTMs during enterovirus infection.

## 1 Introduction

**Figure 1:**
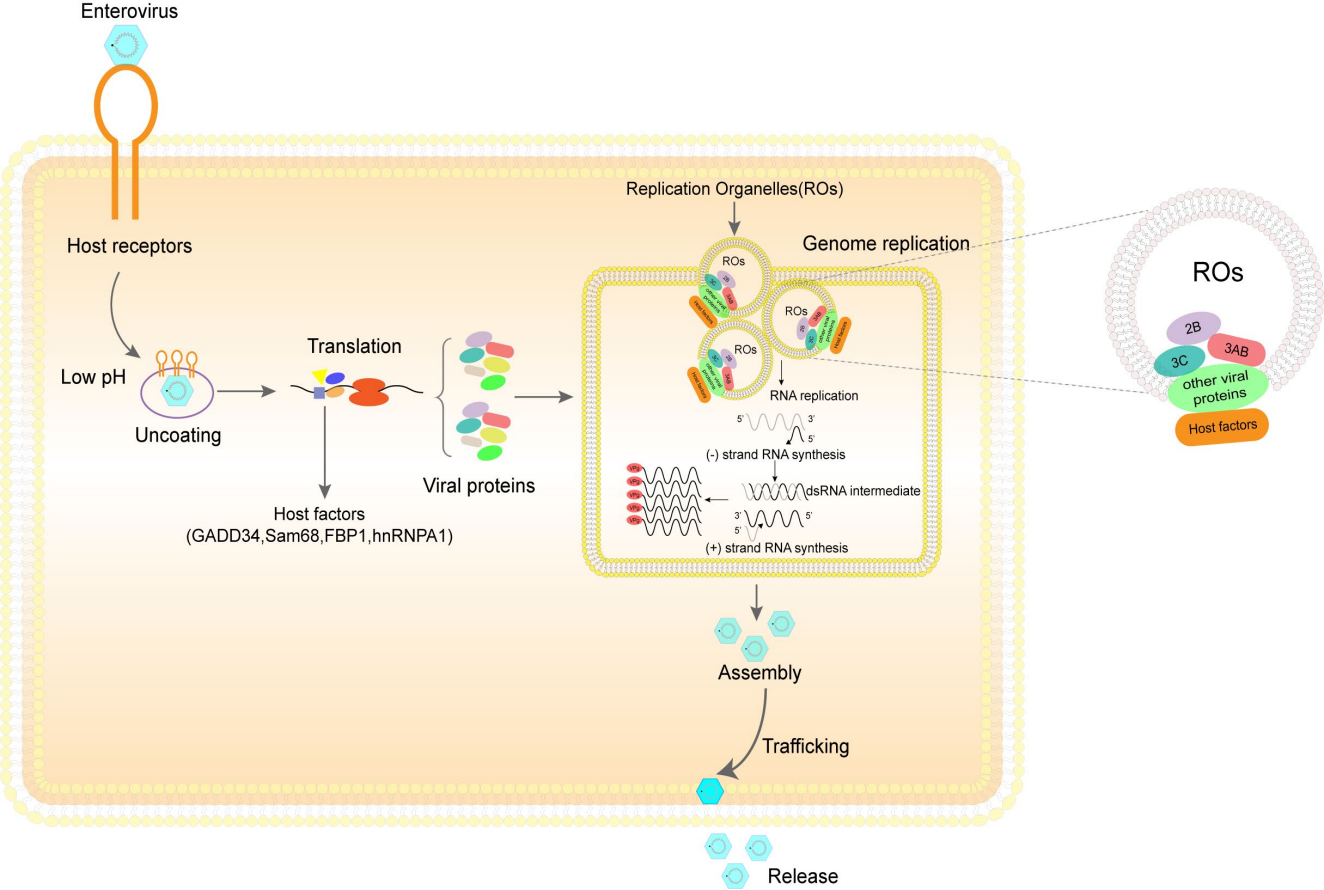
The enterovirus life cycle depends heavily on diverse host machinery. Step 1. Entry: The three main steps by which EV71 virus particles enter the host cell are adhesion onto the host cell surface, binding of viral particles to cell receptors, and entry of viral particles into cells through endocytic pathways. Step 2. Uncoating and genome release: under the influence of the pH environment of the host cell, the virus sheds its shell and releases its genome. Step 3. Translation and cleavage of viral polyprotein: under the influence of various host proteins, translation of viral RNA is initiated and the resulting polyprotein is cleaved to obtain the structural and non-structural proteins. Step 4. Replication of viral genomes: viral genome replication requires the participation of a large number of host factors and viral proteins to complete the formation of replication organelles (ROs) and genome replication. Step 5. Assembly and release of viral particles: the assembly of viral particles also requires coordination between various viral and host proteins. After the assembly, the virus particles are released into the extracellular space in various ways to initiate the next round of infection.

Enterovirus infections caused by various pathogens, such as coxsackievirus B (CVB), poliovirus, and enterovirus 71 (EV71), can cause serious diseases (Bauer et al., 2019; Saguil et al., 2019). To achieve efficient infection, these viruses must co-opt the host cell machinery which is involved in the progression of every stage in the enterovirus life cycle (Figure 1) (Tabor-Godwin et al., 2012; Lyoo et al., 2017; Wu and Chu, 2017). Post-translational modification (PTM) of proteins diversify proteome function by covalently adding small proteins or other groups, potentially altering their cellular localization, interaction with partners, and activation state (Schweppe et al., 2003; Zolg et al., 2018). The disruption of PTMs can upset normal physiological processes (Gupta et al., 2021; He et al., 2021; Smith and Carregari, 2022). The rapid development of proteomics and mass spectrometry has identified an increasing number of PTMs, including glycosylation, ubiquitination, methylation, phosphorylation, acetylation, and lipidation (Aslebagh et al., 2019; Andres et al., 2020; Hermann et al., 2022). The progression of PTMs is dynamic, reversible, and catalyzed by specific enzymes; therefore, many compounds targeting these enzymes can effectively inhibit or promote PTM (Lopez et al., 2016; Sun et al., 2020; Zhu et al., 2022c). Different PTMs on different host and viral proteins can exert various effects on viral growth by targeting viral entry, replication, assembly, egress, and antiviral immune response (Kumar et al., 2020; Song et al., 2021; Cheng et al., 2022; Xue et al., 2022). Here, we review some essential PTMs, including ubiquitination, phosphorylation, acetylation, SUMOylation, tyrosine sulfation, neddylation, and ISGylation, associated with host-pathogen interactions to better understand the role and mechanism of PTMs during enterovirus infection.

## 2 Ubiquitination

As a pivotal PTM, ubiquitination plays a role in various cellular processes in eukaryotes according to egiht differently-linked polyUb chains which established via conjugation to intrinsic ubiquitin residues, including seven Lys residues (Lys6, Lys11, Lys27, Lys29, Lys33, Lys48 or Lys63) or the N-terminal Met1 residue (Chau et al., 1989; Hoege et al., 2002; Lauwers et al., 2009). The differently-linked polyUb chains code for distinct signaling outcomes including cancer development, protein trafficking, cell death, immune response, signaling pathways, and viral infection, through its proteolytic and non-proteolytic functions (Komander, 2009; Dores and Trejo, 2014; van Wijk et al., 2019; Iwai, 2021; Pellegrino et al., 2022; Park et al., 2022a). This process is catalyzed by E1, E2, and E3 which enable ubiquitin (Desai et al.) and Ub-like modifiers, SUMO, NEDD8, and ISG15, to bind to substrate proteins, and is described as ubiquitination, SUMOylation, neddylation, and ISGylation, respectively (Kang and Yi, 2011; Zhang et al., 2017). These modifications play different roles in enterovirus infections.

### 2.1 The antiviral role of ubiquitination in enterovirus infection

**Figure 2:**
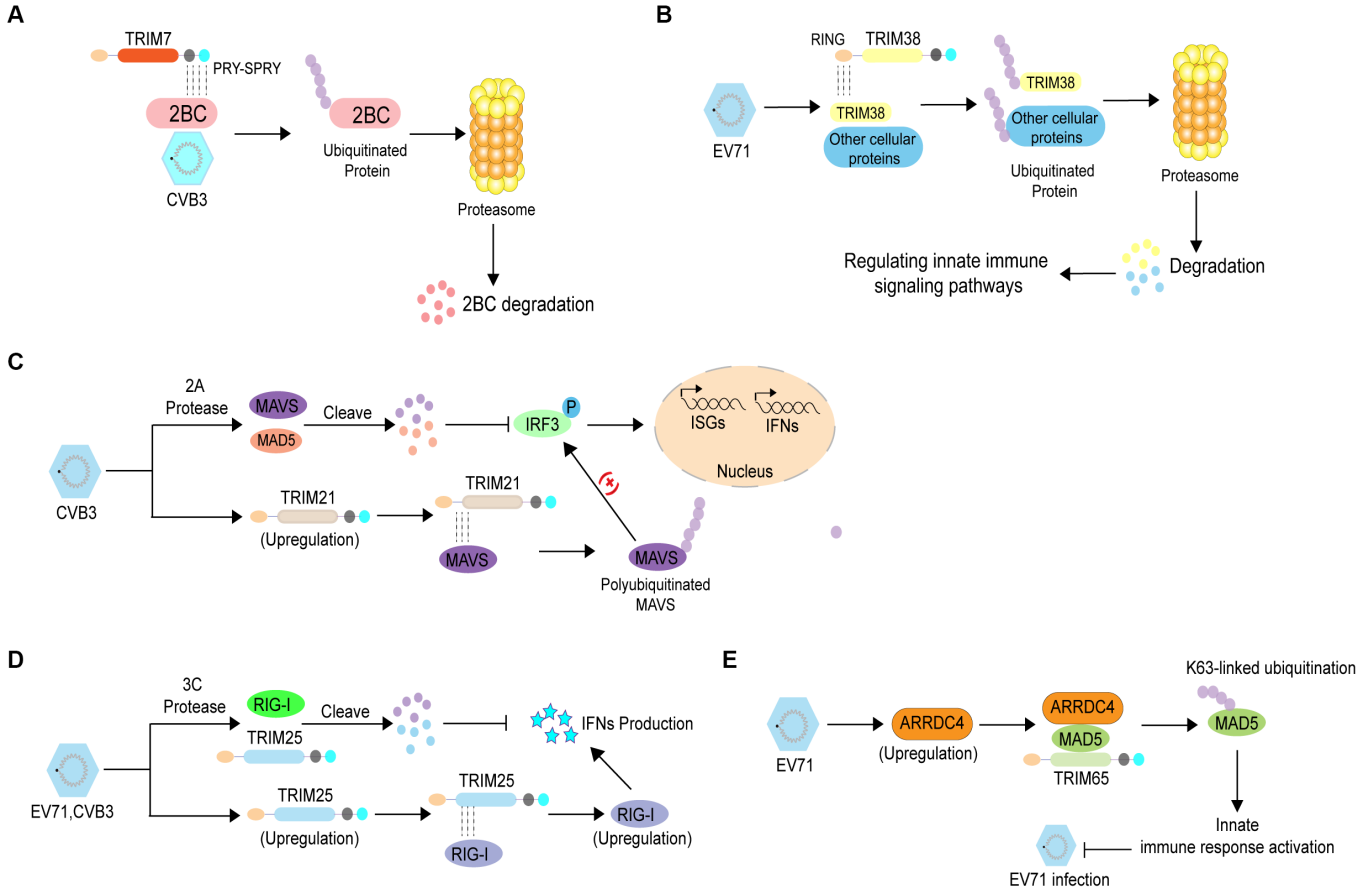
The antiviral role of ubiquitination in enterovirus infection. **(A)** TRIM7 recognizes the C-terminal region of 2C via the PRY-SPRY domain, thereby mediating degradation of the 2 BC (Change 2C into 2 BC) protein to inhibit enterovirus replication. **(B)** TRIM38 disrupts EV71 infection by ubiquitinating cellular proteins that regulate immune signaling pathways or their interactions with viral proteins. **(C)** TRIM21 mediates the enhancement of type I interferon signaling by interacting with mitochondrial anti-viral signaling protein to catalyze the K27-linked polyubiquitination of mitochondrial anti-viral signaling protein, which inhibits CVB3 infection. **(D)** TRIM25 mediates RIG-I ubiquitination and restores RIG-I expression and IFN-β production to prevent enterovirus infection. **(E)** EV71 infection induces ARRDC4 expression Subsequently, ARRDC4 interacts with MDA5 and recruits TRIM65 to increase MDA5 K63 ubiquitination, leading to the activation of the innate signaling pathway; thus, inhibiting EV71 infection.

Ubiquitination is widely involved in various physiological processes, and its dysregulation plays an important role in various diseases, including enterovirus infections (Swatek and Komander, 2016; Rape, 2018). Numerous studies have shown that protein ubiquitination can inhibit enterovirus infection through different mechanisms. An increasing number of studies have indicated that tripartite motif (TRIM) proteins, which have E3 Ub ligase activity, play pivotal roles in inhibiting viral infections including those caused by enteroviruses. A previous study revealed that TRIM7 inhibits enteroviral replication by ubiquitination and degradation of viral 2 BC (Change “2C” into “2 BC”) protein. Mechanistic studies have shown that TRIM7 recognizes the C-terminal region of 2C via the PRY-SPRY domain, thus mediating the degradation of the 2 BC (Change “2C” into “2 BC”) protein (Figure 2A) (Fan et al., 2021). Another study showed that the E3 Ub ligase TRIM38 disrupts EV71 infection by ubiquitinating cellular proteins that regulate immune signaling pathways or interact with viral proteins (Figure 2B) (Liu et al., 2011). In addition, TRIM proteins can suppress enterovirus infection by regulating the immune response through different mechanisms. TRIM21 can mediate the upregulation of type I interferon signaling by interacting with mitochondrial anti-viral signaling protein (MAVS), eventually catalyzing the K27-linked polyubiquitination of MAVS and inhibiting CVB3 infection (Figure 2C) (Liu et al., 2018). Enterovirus 3C proteins suppress retinoic acid-inducible gene I (RIG-I)-mediated type I interferon (IFN) responses via their cleavage activity, however, TRIM25 overexpression rescues this suppression. TRIM25 mediates the RIG-I ubiquitination and restores its expression and IFN-β production, indicating that TRIM25 can abolish enterovirus infection (Figure 2D) (Xiao et al., 2021). Arrestin domain containing 4 (ARRDC4) plays pivotal roles in G-protein-coupled receptor-associated physiological and pathological processes and glucose metabolism. A previous study reported that the expression of ARRDC4 was increased after EV71 infection, both *in vitro* and *in vivo*. Subsequently, ARRDC4 interacts with melanoma differentiation-associated protein 5 (MDA5) and recruits TRIM65 to increase K63-linked ubiquitination of MDA5 (Change “TRIM65 to increase MDA5 K63 ubiquitination” into “TRIM65 to increase K63-linked ubiquitination of MDA5”), eventually leading to the activation of the innate signaling pathway and inhibition of EV71 infection (Figure 2E) (Meng et al., 2017).

The Ub-proteasome system (UPS) can also inhibit enterovirus infection through other mechanisms. The internal ribosome entry site (IRES) is an EV71 genomic functional element that is essential for the translation of viral proteins. Kung YA et al. reported that the negative IRES trans-acting factor KH-type splicing regulatory protein (KHSRP) downregulates IRES-mediated translation in enterovirus-infected cells by interacting with KLHL12, a substrate adaptor for the E3 ligase complex; leading to KHSRP ubiquitination (Table 1) (Kung et al., 2017). Many chemical compounds can also participate in the UPS to regulate enterovirus infections. Curcumin (diferuloylmethane) is a natural polyphenolic compound that dysregulates the UPS. Si X et al. found that curcumin treatment markedly decreases the replication of coxsackieviruses. Further investigation revealed that not only 20S proteasome proteolytic activities but also cellular deubiquitination activities were decreased after curcumin treatment, which led to the accumulation of ubiquitinated proteins and a reduction of free ubiquitin (Change “ubiquitinated protein” into “ubiquitin”) (Table 1) (Si et al., 2007). Pyrrolidine dithiocarbamate (PDTC) is an antioxidant that suppresses ubiquitin proteasome-mediated proteolysis. A previous study reported that the synthesis of CVB3 viral RNA, expression of viral protein VP1, and the release of viral progeny were significantly reduced after PDTC treatment. Further evidence indicated that the negative effect of PDTC on viral infection is probably related to the regulation of ubiquitination (Table 1) (Si et al., 2005b).

**Table 1:** The anti-enterovirus role of other proteins and chemical compounds involved in ubiquitin-proteasome system (UPS).

Proteins and compounds	Mechanisms	Effect	References
KHSRP	KHSRP ubiquitination	Downregulate the IRES-mediated translation in enterovirus infected cells	Kung et al. (2017)
Curcumin	Enhanced accumulation of ubiquitinated proteins	Decreased the replication of coxsackievirus	Si et al. (2007)
PDTC (Pyrrolidine dithiocarbamate)	Regulation of ubiquitination	Reduced CVB3 infection	Si et al. (2005b)

The negative IRES trans-acting factor KHSRP and the chemical compounds curcumin and pyrrolidine dithiocarbamate participate in the UPS to inhibit enterovirus infection.

### 2.2 The proviral role of ubiquitination in enterovirus infection

The ubiquitination of proteins can also play a positive role in enterovirus infection. siRNA-mediated downregulation of Ub can decrease CVB3 infection by downregulating the ubiquitination and degradation of proteins. Furthermore, the 3D protein of CVB3 is modified by ubiquitination, and this ubiquitination is required for efficient viral replication (Table 2) (Si et al., 2008). Additionally, Voss M et al. showed that during CVB3 infection, there is an increase of ubiquitinated (Change “ubiquitinylated” into “ubiquitinated”) proteins in virus-utilized membranes that direct proteins to proteasomal degradation. This process is exploited by CVB3 for the cleavage of viral polyprotein fragments P1 and P3 to ensure correct viral replication (Table 2) (Voss et al., 2021). The host cell cycle regulatory machinery can also be modified by CVB3 to promote viral replication. To create a favorable environment for CVB3 replication, CVB3 infection can disrupt the homeostasis of host cells by increasing the ubiquitin-dependent proteolysis of cyclin D1, which results in cell cycle arrest (Table 2) (Luo et al., 2003). Cardiac failure is a serious complication of CVB3 infections. Intercalated disks (ICDs) are connections that sustain cardiac structures and mediate signal communication among cardiomyocytes, and ICD deficiency can lead to heart dysfunction. Ye X et al. revealed that CVB3-induced miR-21 expression could target the deubiquitinating enzyme YOD1 to increase K48-linked ubiquitination and degradation of desmin, a component of ICDs; resulting in the destruction of desmosomes and enhanced cardiomyocyte injury (Table 2) (Ye et al., 2014). During CVB3 infection, the expression levels of the UPS-related proteins E1A/E1B、UBCH7 (Add“UBCH7”)and UCHL1 are significantly upregulated, indicating that the UPS plays a significant role in CVB3 infection (Table 2) (Gao et al., 2008).

**Table 2:** The proteins and mechanisms involved in ubiquitination promote enterovirus infection.

Proteins and compounds	Mechanisms	Effect	References
siRNA-mediated downreguiation of ubiquitin	Downregulated the ubiquitination and degradation af proteins	Decreased CVB3 infection	Si et al. (2008)
CVB3 3D protein	Modified by ubiquitination	Benefit CVB3 replication	Si et al. (2008)
Prateins at virus-utilized membranes	Processing of the viral polyprotein fragments P1 and P3	Benefit CVB3 replication	Voss et al. (2021)
Cyclin D1	Increasing ubiquitin-dependent proteolysis af cyclin D1	Benefit CVB3 replication	Luo et al. (2003)
Desmin	K48-linked ubiquitination and degradation of desmin	Enhance the injury of CVB3-infected cardiomyocytes	Ye et al. (2014)
E1A/E1B, UBCH7, UCHL1	The expression level of E1A/E1BUBCH7 and UCHL1 enhanced	Benefit CVB3 replication	Gao et al. (2008)

siRNA-mediated downregulation of ubiquitin, higher levels of ubiquitinated (Change “ubiquitinylated” into “ubiquitinated”) proteins, increased ubiquitin-dependent proteolysis of cyclin D1, YOD1-mediated increase in desmin K48-linked ubiquitination, and UPS-related proteins E1A/E1B and UCHL1 all play proviral roles during enterovirus infection through different mechanisms.

**Figure 3:**
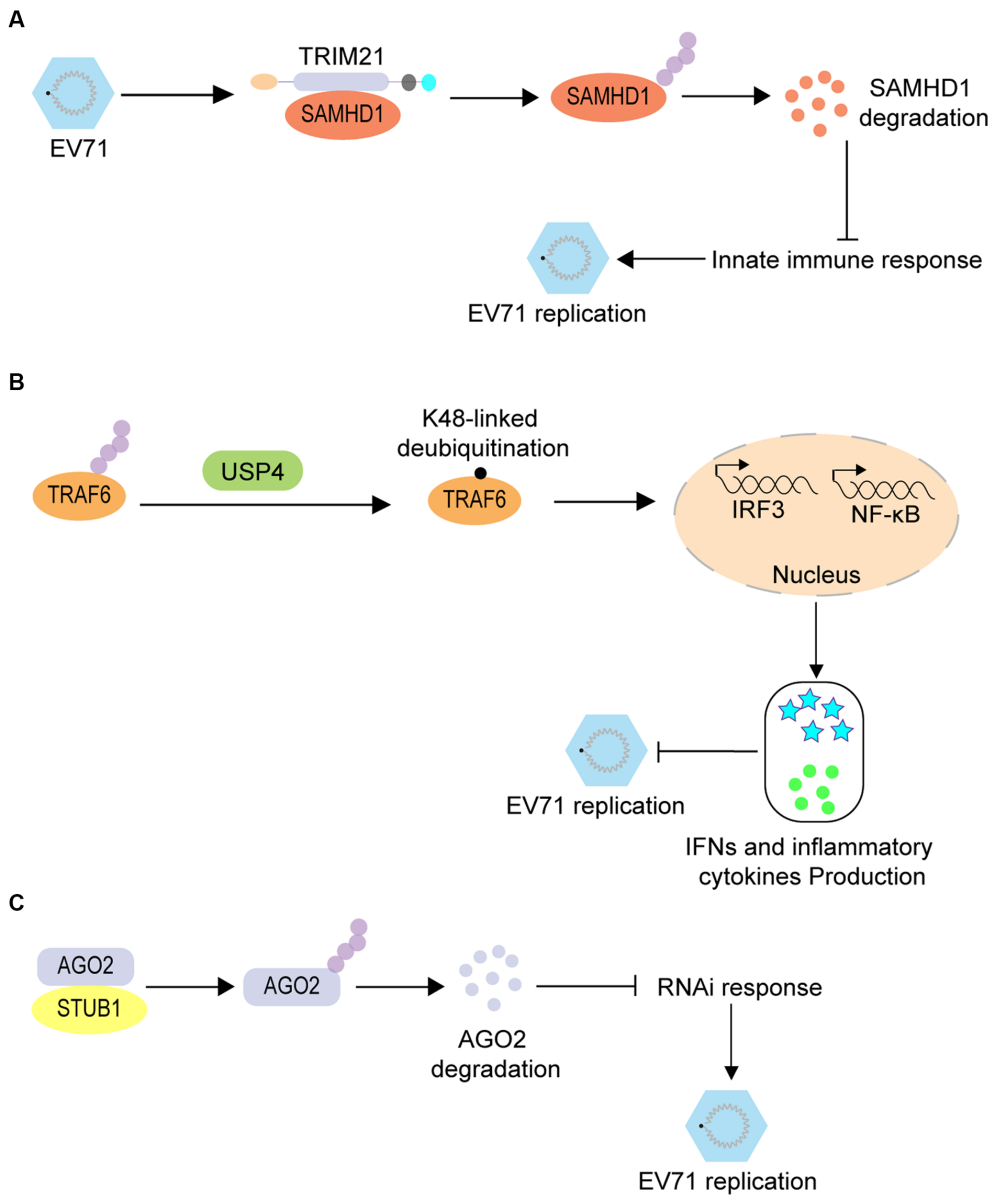
The proviral role of ubiquitination in enterovirus infection. **(A)** TRIM21-mediated ubiquitination of SAMHD1 leads to the degradation of SAMHD1, thus promoting EV71 infection. **(B)** USP4-mediated TRAF6 K48-linked deubiquitination induces the upregulation of RLR-induced NF-κB signaling to suppress the EV71 replication. **(C)** STUB1 interacts with AGO2 and accelerates the K48-linked ubiquitination of AGO2, thus promoting its degradation and decreasing the RNAi response; thereby promoting EV-71 replication.

Similarly, ubiquitination promotes enterovirus infection by regulating immune responses. SAM and HD Domain containing Deoxynucleoside Triphosphate Triphosphohydrolase 1 (SAMHD1) is an effector of innate immunity and has been confirmed as a restrictive factor for EV71. However, further studies have revealed that during EV71 infection, this inhibition can be overcome via ubiquitination of SAMHD1 by TRIM21 and its subsequently degradation through the proteasomal pathway (Figure 3A) (Li et al., 2020). Tumor necrosis factor receptor-associated factor 6 (TRAF6) is an important protein in the RIG-I-like receptor (RLR)-mediated antiviral signaling pathway that also regulates EV71 infection via a ubiquitination-associated pathway. RLR-induced NF-κB signaling is upregulated by ubiquitin-specific protease 4 (USP4)-mediated TRAF6 K48-linked deubiquitination, which ultimately suppresses EV71 replication (Figure 3B) (Xu et al., 2018). RNA interference (RNAi) is a conserved antiviral immune mechanism in various eukaryotes, and STIP1 homology and U-box containing protein 1 (STUB1) regulates the RNAi machinery in mammals. A previous study revealed that STUB1 interacts with Argonaute RISC Catalytic Component 2 (AGO2) and accelerates its K48-linked ubiquitination; thus facilitating its degradation, decreasing the RNAi response, and promoting EV71 replication in mammalian cells (Figure 3C) (Zhang et al., 2022).

### 2.3 The roles of Ub-like modification in enterovirus infection

Both of SUMO, NEDD8 and ISG15 are Ubiquitin-Like Proteins (Herrmann et al., 2007). The SUMO proteins share structural similarities with ubiquitin, and the conjugation of SUMO proteins to substrates happens via a serious of enzymatic cascade involving the E1,E2 and E3 protein ligase. When a SUMO peptide tagged on the lysine residue of the protein substrate it could regulated various cellular processes, including transcription, replication, chromosome segregation and DNA repair (Geiss-Friedlander and Melchior, 2007; Gareau and Lima, 2010). NEDD8 is 60% identical and 80% homologous to ubiquitin. When NEDD8 tagged to the substrate protein, it regulated protein metabolism and activity (Cappadocia and Lima, 2018). Interferon-Stimulated Gene 15 protein (ISG15) is expressed at low levels under physiological conditions (Desai et al., 2006). Compared with ubiquitin, ISG15 shows substantial sequence variation from species to species. Once ISG15 is tagged to targets within the cell upon interferon stimulation, these cellular targets are involved in every aspect of cellular function, including DNA replication/repair, cell metabolism, signal transduction, and cytoskeletal organization and others (Mirzalieva et al., 2022).

**Figure 4:**
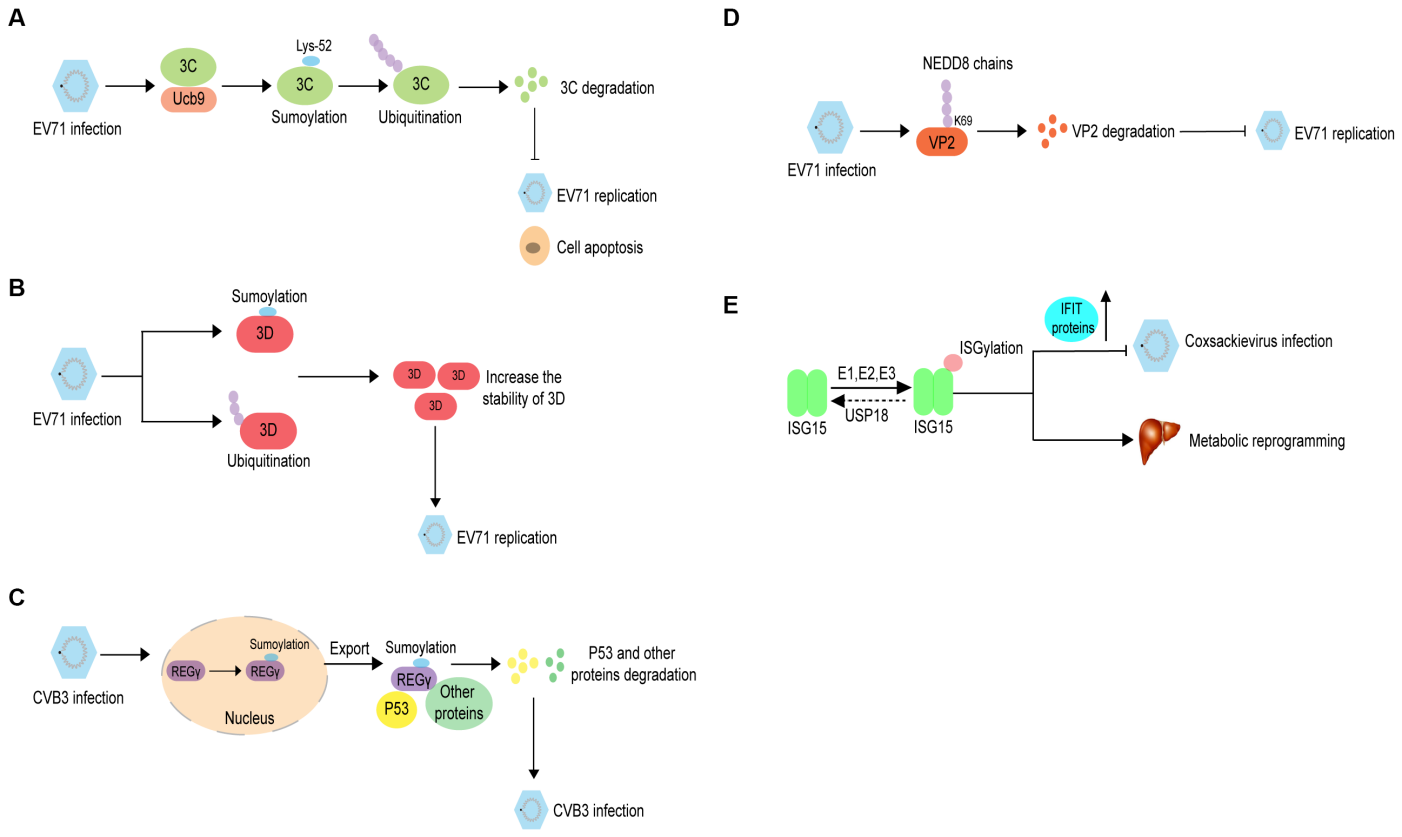
The roles of Ub-like modification in enterovirus infection. **(A)** EV71 3C protein can be SUMO-modified by Ubc9 which decreases the protease activity and protein stability of 3C, thereby decreasing EV71 replication. **(B)** SUMOylation of the EV71 3D protein cooperates with 3D ubiquitination to increase its stability, eventually promoting the replication of EV71. **(C)** SUMOylation of REGγ causes it to translocate from the nucleus to the cytoplasm, allowing the possible interaction of REG with viral or host proteins to play a proviral role during CVB3 infection. **(D)** The VP2 protein of EV71 is modified by NEDD8 at lysine 69, reducing its stability and decreasing viral replication. **(E)** Protein ISGylation blocks coxsackievirus pathology by increasing antiviral effectors, IFIT1/3 proteins, and metabolic reprogramming.

Viral proteins can be SUMOylated during the enterovirus life cycle. EV71 3C protein can be SUMO-modified by Ubc9, which decreases its protease activity and protein stability; thus decreasing EV71 replication (Figure 4A) (Chen et al., 2011). In addition, the 3D protein is modified by SUMO-1 during infection and combined with 3D ubiquitination, this can increase the stability of the 3D protein which promotes EV71 replication (Figure 4B) (Liu et al., 2016).

Host proteins can also be SUMOylated during viral infection. The SUMOylation of REGγ, a component of the 11S proteasome activator, causes it to translocate from the nucleus to the cytoplasm, offering possibilities for the interaction of REG with viral or host proteins to play a proviral role during CVB3 infection (Figure 4C) (Gao et al., 2010). Neddylation also affects enterovirus infection. The VP2 protein of EV71 can be modified by NEDD8 at lysine 69, which reduces VP2 stability and decreases viral replication (Figure 4D) (Wang et al., 2022). Several studies have shown that protein modification with ISG15, referred to as ISGylation, plays a pivotal role in type I IFN-induced antimicrobial systems. One study revealed that ISGylation blocks coxsackievirus pathology by increasing the levels of antiviral effector IFIT1/3 proteins and metabolic reprogramming (Figure 4E) (Kespohl et al., 2020).

## 3 Phosphorylation

Phosphorylation is a reversible process involving protein kinases and phosphatases which catalyze the phosphorylation of serine, threonine, and tyrosine in proteins. Protein phosphorylation plays a broad role in various biological events, including protein stability, protein interactions, transcription regulation, signal transduction, intracellular localization, and cell cycle progression (Zhao et al., 2010; Zhang and Pelech, 2012; Deutscher et al., 2014; Puertollano et al., 2018; Yamasaki et al., 2020; Bilbrough et al., 2022; Park et al., 2022b). In addition, phosphorylation of both viral and cellular proteins can significantly affect enterovirus pathogenesis.

### 3.1 The antiviral role of phosphorylation in the infection of enterovirus

Currently, there are relatively few reports on inhibition of protein phosphorylation during enterovirus infection. One study revealed that the natural compound emodin, a natural compound derived from plant roots, can inhibit CVB3 replication by suppressing Akt/mTOR signaling and activating 4EBP1 and eEF2K (Table 3) (Zhang et al., 2016). Curcumin is a compound obtain anti-cancer properties that can decrease EV71 infection by inhibiting the phosphorylation of PKCδ (Table 3) (Huang et al., 2018). Additionally, protein phosphorylation plays an antiviral role in enterovirus infection via an immune-regulated pathway. A study revealed that G3BP1 plays an anti-enterovirus role by inducing stress granule formation, and is associated with the innate immune transcriptional response activation through NF-κB and JNK (Table 3) (Reineke and Lloyd, 2015). Manassantin B inhibits CVB3 replication by activating the STING/TKB-1/IRF3 antiviral pathway and increasing the production of mROS (Song et al., 2019). Cathelicidin antimicrobial peptides (human LL-37 and mouse CRAMP) (Add “human LL-37 and mouse CRAMP”) also play an antiviral role in EV71 infection and (Delete”and”) LL-37 and CRAMP were shown to markedly increase the basal IFN-β expression and IRF3 phosphorylation, effectively alleviating EV71 infection (Yu et al., 2021). Trehalose prevents cardiovascular diseases by regulating autophagy. In a viral myocarditis mouse model induced by CVB3, trehalose was shown to reduce myocardial injury and significantly enhance AMPK and ULK1 phosphorylation in B cells (Wei et al., 2022). During EV71 infection, STAT3 phosphorylation and the expression of downstream inflammatory regulators are increased, which activates the type I IFN-mediated antiviral response (Wang et al., 2019). In addition, the host IKKε gene leads to alterations in IFN production during EV71 infection through phosphorylation and translocation of IRF7 in the presence of ubiquitin, activating the expression of IFNβ and ISGs and attenuating viral propagation (Table 3) (Chang et al., 2021).

**Table 3:** The antiviral role of phosphorylation in enterovirus infection.

Proteins and compounds	Mechanisms	Effect	References
Emodin	Suppression Akt/mTOR signalling and activation 4EBP1 and eEF2K	Suppress CVB3 replication	Zhang et al. (2016)
Curcumin	The phosphorylation of PKCδ	Decrease EV71 infection	Huang et al. (2018)
G3BP1	Activation of innate immune transcriptional responses through NF-κB and JNK	Against enteroviruses infection	Reineke and Lloyd (2015)
Manassantin B	Activation of the STING/TBK-1/IRF3 antiviral pathway	Inhibit CVB3 replication	Song et al. (2019)
Cathelicidin	increased the basal lFN-β expression and IRF3 phosphorylation	Inhibit EV71 infection	Yu et al. (2021)
Trehalose	Phosphorylation of AMPK and ULK1	Reduce myocardial injury in CVB3-infected mice	Wei et al. (2022)
STAT3	STAT3 phosphorylation	Inhibit EV71 intection	Wang et al. (2019)
IKKε	Phosphorylation and translocation of IRF7	Against EV71 infection	Chang et al. (2021)

### 3.2 The proviral role of phosphorylation in enterovirus infection

Phosphorylation also plays an important role in the promotion of enterovirus infection. The cellular phosphoproteome undergoes significant changes and approximately 85% of the quantified phosphoproteome is dynamically regulated during CVB3 infection (Giansanti et al., 2020). Once the EV71 virus binds to host receptors, the phosphorylation of PI3K/Akt and MAPK/ERK is initiated; immediately inactivating GSK3β, delaying host cell apoptosis, and promoting infection (Wong et al., 2005). The IRE1/XBP1 pathway plays a pivotal role in the endoplasmic reticulum (ER), or unfolded protein, stress response. One study revealed that XBP1 participates in EV71 replication by affecting viral entry. After EV71 infection, IRE1 undergoes phosphorylation and activation, whereas the downstream XBP1s (spliced XBP1) protein levels decreased (Jheng et al., 2012). During poliovirus and CVB3 infection, IRE1 undergoes the complicated dynamics of autophosphorylation and cleavage, indicating that enteroviruses utilize various mechanisms to regulate the Ire1-Xbp1 host defensive pathway in infected cells (Shishova et al., 2022) (Table 4).

**Table 4:** The proviral role of phosphorylation in the infection of enterovirus.

Proteins and compounds	Mechanisms	Effect	References
GSK3β	Phospharylation of Pl3K/Akt and MAPK/ERK	Promote EV71 infection	Wong et al. (2005)
IRE1/XBP1	The phosphorylation of IRE1	Promote enteroviruses propagation	Jheng et al. (2012) and Shishova et al. (2022)
PSF (PTB associated splicing factor)	PSF phosphorytation	Benefit CVB3 RNA translation	Dave et al. (2017)
RASSF4	Inhibit AKT phosphorylation	Promote EV71 replication	Zhang et al. (2015)
Vimentin	Vimentin phosphorylation and aggresomes formation	Promote EV71 replication	Haolong et al. (2013)
c10orf76	Phosphorylation of PI4KB	Promote enteroviruses replication	McPhail et al. (2020)
GRP78/BiP	Phosphorylation of PKR	Promote EV71 replication	Jheng et al. (2016)
EV71 3C protein	PKR phosphorylation	Promote EV71 replication	Chang et al. (2017)
Sam68	PI3K/Akt activation	Benefit EV71 infection	Zhang et al. (2014)
Protein Kinase C	The localization of phosphorylated PKC	Cell susceptibility to Echovirus1 infection	Turkki et al. (2013)
Abl kinase	Abl kinase activation	Promote the CVBs entry and RNA release	Coyne and Bergelson (2006)
Fyn kinase	Fyn kinase activation and phospharylation of caveolin	Promote CVBs entry	Coyne and Bergelson (2006)
SAPKs (Stress-activated prokein kinases)	Phosphorylation of JNK1/2 and p38 MAPK	Promote CVB3 progeny release	Si et al. (2005a)
HSF1	HSF1 phosphorylation	Stabilize the genome of CVB3	Qiu et al. (2016)
Vimentin	Adivation of NLRP3 by VIM (Vimentin)-ERK-NF-κB pathway	Benefit EV71 infection	Gong et al. (2022)
SOCS proteins	Inhibiting STAT3 phosphorylation	Promote EV71 infection	Gao et al. (2020)
EV71 2C Protein	Suppressing lkkβ phosphorylation	Benefit EV71 infection	Zheng et al. (2011)
Bosentan	p38 MAPK phosphorylation	Facilitate the progression of Caxsackievirus Induced Myecarditis	Marchant et al. (2009)

The phosphorylation of many proteins can regulate stages in the life cycle of enteroviruses. PTB associated splicing factor (PSF) is an IRES trans-acting factor which is critical for CVB3 RNA translation. One study revealed that PSF protein levels in the cytoplasm are enhanced during CVB3 infection and its phosphorylation promotes CVB3 RNA translation (Dave et al., 2017). The tumor suppressor RASSF4, which participates in diverse biological processes including cell death, signal transduction, and tumor development, has been reported to promote EV71 infection and subsequently accelerate the inhibition of AKT phosphorylation in infected cells (Zhang et al., 2015). The type III intermediate filament vimentin has been reported to be a key factor in the process of viral infection through different mechanisms. In EV71 infection of human astrocytoma cells, vimentin undergoes rearrangement immediately following the formation of aggresome-like structures in the perinuclear region. Further research has indicated that vimentin is phosphorylated, and that both phosphorylation and aggresome formation are important for EV71 replication (Haolong et al., 2013) (Table 4).

Enterovirus replication is heavily reliant on PI4KB kinase activity, and the interaction between PI4KB and c10orf76 is a critical Golgi signaling complex and an important factor influencing the replication of several enteroviruses. The affinity between PI4KB and c10orf76 is determined by the phosphorylation status of PI4KB, and the phosphorylated PI4KB at Ser496 site shows a decrease in affinity for c10orf76 (McPhail et al., 2020). Enteroviruses can also use ER stress or the unfolded protein response to promote viral replication. Jheng JR et al. revealed that ER stress and the unfolded protein response, GRP78/BiP, were redistributed during EV71 infection, which contributed to the promotion of EV71 replication. Further studies showed that dsRNA, rather than a viral protein, induces the phosphorylation of PKR, which plays a role in GRP78/BiP redistribution (Jheng et al., 2016). However, another study showed that in addition to dsRNA, viral proteins can also cause PKR phosphorylation, and that the EV71 3C protein interacts with PKR and subsequently mediates its phosphorylation to promote viral replication (Chang et al., 2017) (Table 4).

Numerous studies have shown that nuclear proteins translocate into the cytoplasm to promote EV71 replication. During EV71 infection, Sam68 translocates to the cytoplasm and mediates the activation of PI3K/Akt to assist the infection (Zhang et al., 2014). The localization of phosphorylated host proteins is related to the susceptibility to viral infections. In a study of cell susceptibility to Echovirus1, phosphorylated PKC (Change “pPKC” into “phosphorylated PKC”) was mainly distributed in the cytoplasm and usually accumulated in the perinuclear compartment in non-permissive cells, whereas in permissive cells, it was evenly distributed and no perinuclear aggregation was observed (Turkki et al., 2013) (Table 4).

The activation of some kinases also plays a significant role in EV71 infection. The process of virus attachment to decay-accelerating factor on the surface of apical cells activates Abl kinase, which promotes the movement of the virus to tight junctions where it immediately interacts with coxsackievirus-adenovirus receptor, facilitating the alteration of conformation in the viral capsid. This process is critical for entry and RNA release. Additionally, interaction with decay-accelerating factor can activate Fyn kinase, which is necessary for caveolin phosphorylation and viral transportation into cells (Coyne and Bergelson, 2006). Another study reported that the phosphorylation of JNK1/2 and p38 MAPK was enhanced during CVB3 infection and that the stress-activated protein kinase pathway plays a critical role in the life cycle of CVB3, especially during viral progeny release (Si et al., 2005a). CVB3 infection induces phosphorylation of heat shock factor 1, leading to the upregulation of Hsp70-1 which contributes to the stabilization of the CVB3 genome; thus promoting viral infection (Qiu et al., 2016) (Table 4).

Phosphorylation also affects the host immune system, promoting EV infection, and the occurrence and development of enterovirus-related diseases. The inflammasome is an essential element of the natural immune system and is closely associated with EV71-induced central nervous system injury, which is regulated by signaling pathways such as VIM (Vimentin)-ERK-NF-κB pathway (Add “Vimentin”) (Gong et al., 2022). Viruses can evade the host immune response by increasing the expression of suppressor of cytokine signaling proteins. One study revealed that suppressor of cytokine signaling 3 promotes EV71 infection by inhibiting interferon-induced STAT3 phosphorylation and negatively regulates the JAK/STAT signaling pathway; thus, allowing EV71 to escape host immunity (Gao et al., 2020). Viral proteins also participate in immune responses related to phosphorylation. For example, EV71 2C protein inhibits TNF-α–mediated activation of NF-κB by suppressing Ikkβ phosphorylation (Zheng et al., 2011).

In addition to endogenous cellular phosphorylation, chemical drugs increase the phosphorylation of cellular proteins to regulate viral infections. The endothelin-1 receptor antagonist Bosentan can facilitate the progression of Coxsackievirus-induced myocarditis by inducing p38 MAPK phosphorylation, which increases the viral load in cells and tissues (Marchant et al., 2009) (Table 4).

## 4 Acetylation

Protein acetylation is the principal PTM catalyzed by acetyltransferases (Baeza et al., 2016). Protein acetylation affects numerous biological events, including gene replication, transcription, repair, and signal transduction pathways; thereby regulating diverse cellular processes (Arnesen, 2011; Verdin and Ott, 2015). Host and viral proteins undergo acetylation which can play pivotal roles in different phases of viral infection, including viral entry, genome replication, assembly and release of progeny viral particles, and host antiviral responses (Murray et al., 2018; Xue et al., 2022). With the rapid development and optimization of proteomics and mass spectrometry technologies, a new era of protein acetylation research during viral infection has recently emerged.

### 4.1 The antiviral role of acetylation in the infection of enterovirus

**Figure 5:**
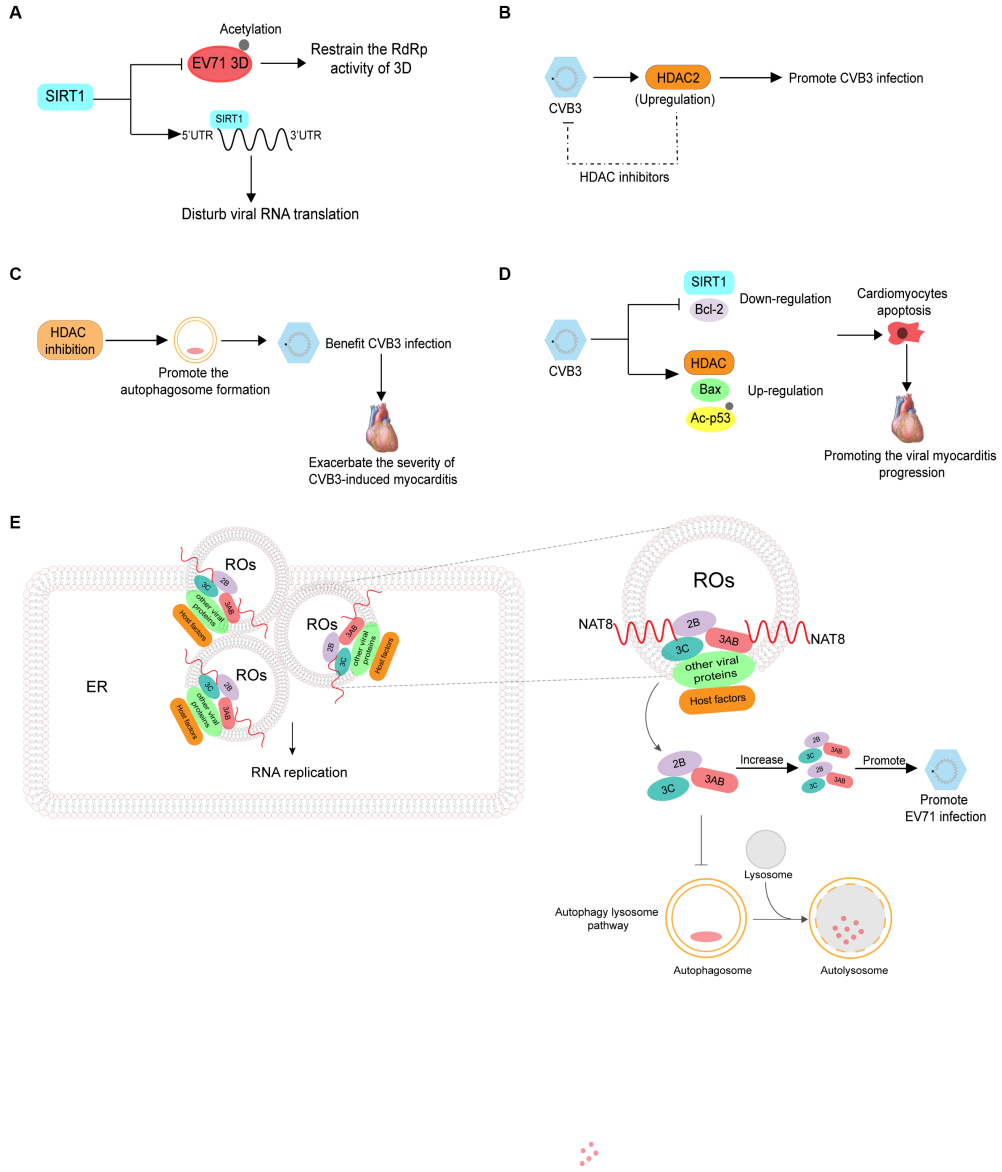
The roles of acetylation in the infection of enterovirus. **(A)** SIRT1 can inhibit the acetylation and RNA dependent RNA polymerase activity of 3D pol, thus reducing viral genome replication. SIRT1 also interacts with the 5′ UTR of EV71 RNA to disrupt viral RNA translation. **(B)** CVB3 infection induces histone deacetylase2 (HDAC2) activity, and treatment with HDAC inhibitors can inhibit CVB3 replication. **(C)** Inhibition of HDAC activity increases the formation of autophagosomes which promote CVB3 replication and ultimately exacerbate the severity of CVB3-induced myocarditis. **(D)** CVB3 infection induces the expression of HDAC1 and Bax while suppressing SIRT1 and Bcl-2, in addition to upregulating acetylated p53. **(E)** NAT8 promotes EV71 replication by increasing the stability of 2B, 3AB, and 3C proteins in an acetyltransferase-activity-dependent manner.

Sirtuin 1 (SIRT1) is a lysine deacetylase that regulates various processes including inflammation, metabolism, and aging. Han Y et al. revealed that reactive oxygen species generation and SIRT1 expression were downregulated in apoptotic cells infected with EV71, and treatment with a reactive oxygen species inhibitor decreased EV71 propagation and increased SIRT1 expression in EV71-infected cells. SIRT1 can inhibit the acetylation and RNA dependent RNA polymerase activity of 3D pol, thereby decreasing viral genome replication. Additionally, SIRT1 can interact with the 5′ UTR of EV71 RNA to disrupt viral RNA translation (Figure 5A) (Han et al., 2016). CVB3 infection induces HDAC2 activity and treatment with a HDAC inhibitor can inhibit CVB3 replication, indicating that the acetylation of proteins can suppress viral infection (Figure 5B) (Shim et al., 2013). There are relatively few studies on inhibition of enterovirus infection by acetylation and further research is required to fully understand this process.

### 4.2 The proviral role of acetylation in enterovirus infection

Contrary to the results above, Zhou L et al. revealed that inhibition of HDAC can increase autophagosome formation, which benefits CVB3 replication and ultimately exacerbates the severity of CVB3-induced myocarditis (Figure 5C) (Zhou et al., 2015). In addition to promoting viral infections, acetylation is related to the pathogenesis of viral myocarditis. CVB3 infection has been reported to induce the expression of HDAC1 and Bax while suppressing SIRT1 and Bcl-2 and upregulating acetylated p53. These events lead to cardiomyocyte apoptosis, thus promoting viral myocarditis progression (Figure 5D) (Jiang et al., 2019). NAT8 is an ER-resident acetyltransferase and promotes EV71 replication by increasing the stability of 2B, 3AB, and 3C proteins in an acetyltransferase activity-dependent manner (Figure 5E) (Zhao et al., 2022).

## 5 Conclusion

PTM of proteins is involved in various biological events, and disruption of the modification process can lead to the development of diseases (Chaugule and Walden, 2016; Caruso Bavisotto et al., 2020; Morales-Tarre et al., 2021; Zhu et al., 2022b). PTM participates in diverse viral infections, including hepatitis B, influenza, SARS-CoV-2, and enterovirus (Karim et al., 2020; Hofmann et al., 2023; Liang et al., 2023; Park et al., 2023). Currently, over 200 distinct covalent modifications have been reported; with phosphorylation, ubiquitination, acetylation, glycosylation being among the most common (Tsikas, 2021). A growing body of work suggests that different PTMs play pro- or antiviral roles through different mechanisms, such as altering the abundance and biochemical properties of host and viral proteins, reducing proteins/protein or proteins/nucleic acid interaction, and regulating innate and adaptive immune responses. Although many studies have confirmed the roles of PTMs in viral infection, the understanding of PTMs has been limited by traditional approaches, owing to the difficulties in detection and complexity of these modifications (Dai et al., 2021; Zhu et al., 2022a). Traditionally, researchers utilize antibody-based assays to reveal specific PTMs; however, this detection approach fails to confirm the combinatorial patterns of diverse PTMs, and the antibodies have difficulty distinguishing specific modification sites (Hattori and Koide, 2018; Leutert et al., 2021). In recent years, increasing evidence of crosstalk between PTMs also has been reported (Cuijpers and Vertegaal, 2018; Vu et al., 2018; Kirsch et al., 2020; Leutert et al., 2021). Therefore, further in-depth investigation of the role and mechanisms of PTM crosstalk and other modifications in enterovirus infection is required.

## Author contributions

XZ: Writing – original draft, Investigation, Software, Supervision, Writing – review & editing. YH: Writing – original draft. JZ: Writing – original draft. YL: Writing – original draft. XM: Writing – review & editing. HC: Writing – review & editing. YX: Writing – review & editing.
